# Enrolling Adolescents in Research on HIV: Young Women Must Be Included

**DOI:** 10.1371/journal.pmed.0030537

**Published:** 2006-12-26

**Authors:** Alana de Kock, Stephanie Skoler, Khatija Ahmed, Sumen Govender

As researchers managing an HIV prevention microbicide trial, which takes place across three provinces, we would like to commend Singh et al. for their informative article on the need to include adolescent women in HIV prevention trials [1]. This commendation is based on our clinical trial experience where we have enrolled 16- and 17-year-old women and are actively continuing with their follow-up.

In our study, we include these young women, who are often those at highest risk for HIV infection, for several reasons. Most notable among these are:

(1) All participants in HIV prevention trials (regardless of the arm to which they are randomised) have a reduced risk of HIV infection, due to the intervention of preventive counselling and education that is ethically required for participants in such trials. In addition, access to sexually transmitted infection (STI) diagnosis and treatment and referrals for partner treatment while in the clinical trial further serves to reduce risk factors for HIV infection and STI re-infection.
(2) These young women are sexually active and at risk, and form part of the end-user constituency for a proven microbicide. This sexual activity is evidenced by statistics from the Human Sciences Research Council for 2005 for South Africa, which show that the prevalence of HIV in the 15- to 34-year-old group is 20%. Furthermore, the Children's Bill passed in June 2005 [2] says that young women can have access to contraception from the age of 12 years without parental consent. It is thus ethically imperative that young women should be included in the testing of such products.
(3) Including those currently at greatest risk for infection ensures that the study will be able to detect the efficacy of the intervention.

It is clear that both the participants and the research question benefit from the inclusion of younger women in communities where HIV risk is high and education, counselling, and new prevention technologies are needed.

Younger women are most at risk when it comes to sexual transmission of HIV [3]. Clinical HIV prevention trials provide an environment where confidentiality can be maintained and where young women can be educated on methods to protect themselves in a confidential and consistent manner through trial participation. Thus, the inclusion of young women in such trials is an opportunity to empower the younger generation of young women across communities. To deny that these young women are sexually active would be to turn a blind eye to an important epicentre of the epidemic, and counterproductive to the HIV prevention research agenda.

The age of consent for adolescent participation in clinical trials without obtaining legal guardian/parental support is a major factor that complicates their involvement in HIV prevention trials and other health-care research. The requirement for parental consent that often accompanies the ethical approval process of most studies is at times an insurmountable barrier and distances the young women that require this type of intervention from securing access to these potential prevention options. As Singh et al. point out, there are many salient reasons why adolescents can and should be considered capable of giving informed consent without parental assent or approval. Researchers must continue to work in collaboration with local and scientific communities, ethics committees, and policy makers to ensure that the need for the participation of young women in HIV prevention trials is understood. It would seem then that both researchers and ethicists should work with actual examples of ethical research activities that include adolescents without parental involvement, in order to develop guidelines for adolescent research in the field of HIV that are both protective and practical. 3
